# Rudolph, the kids’ ward reindeer: a scoping review of the effects of support animals on the well-being of healthcare staff

**DOI:** 10.1186/s12995-023-00395-1

**Published:** 2023-12-12

**Authors:** Thomas C. Erren, Jonas Wallraff, Ursula Wild, David M. Shaw, Philip Lewis

**Affiliations:** 1 Institute and Policlinic for Occupational Medicine, Environmental Medicine and Prevention Research, University Hospital of Cologne, Cologne, Germany; 2 Department of Health, Ethics and Society, CAPHRI Research Institute, Maastricht University, Maastricht, Switzerland; 3 Institute for Biomedical Ethics, University of Basel, Basel, Switzerland

**Keywords:** Support animal, Healthcare staff, Animal assisted activity, Animal assisted therapy

## Abstract

**Background:**

Many systematic reviews identify support animals or animal assisted activity as a beneficial and standard practice in several medical disciplines for patients (children, adolescents, and adults) and residents in care homes. A variety of animals are used such as dogs, cats, ponies, horses, alpacas, reindeer, penguins, rabbits, and tarantulas. Our objective was to explore the evidence regarding effects of animal assisted activity on a further population of interest; namely, healthcare staff.

**Methods:**

We asked the question “how do support animals in healthcare settings affect the well-being of healthcare staff?” As an addendum, we were also interested in what - possibly more unique - animals have visited healthcare settings at Christmas time in particular. We conducted a scoping literature review using PubMed and Web of Science (search as of 26 April 2023).

**Results:**

Twenty studies (in the USA, Australia, Europe; dogs: *n* = 19; cats: *n* = 1) since 2002 included: studies with biological measures (*n* = 3), longitudinal survey studies with analyses (*n* = 5), cross-sectional survey studies with analyses (*n* = 2), and cross-sectional survey studies with descriptive statistics (*n* = 10). Overall, animal assisted activities appear to be well-received by staff and there do not seem to be negative impacts on staff well-being.

**Conclusions:**

Relevant positive effects and avenues of research are identified. Our review suggests that, but not exactly how, animal assisted activity benefits staff. Study evidence is limited with most studies being cross-sectional, descriptive, having low participant numbers, and mostly only involving dogs. Nonetheless, the evidence is mostly positive. The potential of animal assisted activities impacting positively on staff well-being warrants systematic research. Gaps in hard-fact-evidence should not deter us – especially at the festive season – to encourage work with, and systematic research regarding, support animals that provide warmth, empathy, comfort, and more in healthcare settings.

## Background

**Fig. 1:**
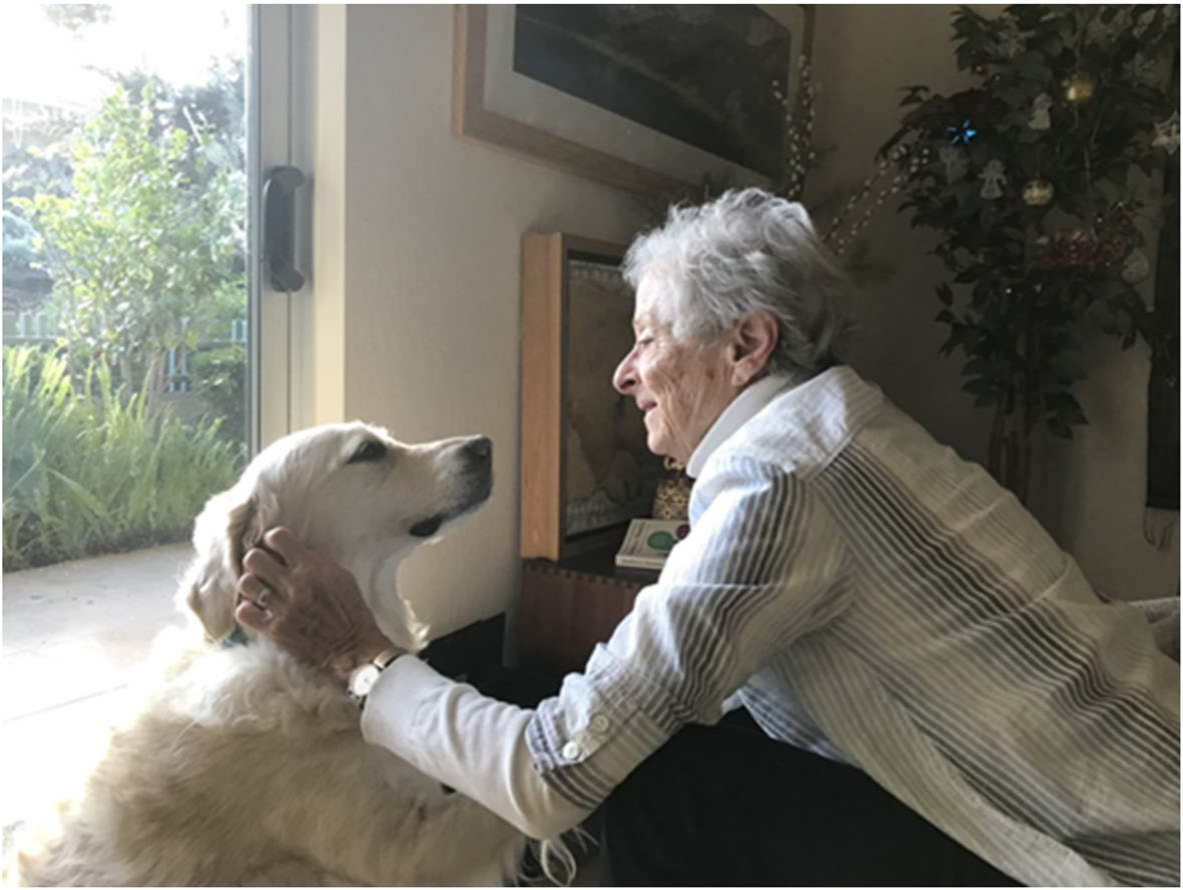
Maya, like many Golden Retrievers, loves to socialize with humans and seems to get as much pleasure from meeting humans as they do in meeting Maya. So it is a mutually therapeutic encounter. Published with permission from Raymond Neutra (photography)

In the 1950s, the father of one of the authors organized for a baby elephant to visit a children’s ward in Bonn (Germany) for Christmas. At that time, this opportunity was afforded with the help of a small visiting circus. The young elephant, teamed up with a clown [1, 2], contributed to special Christmas days for the children in the hospital. To-date, a whole range of – more or less unusual – animals have been welcome guests in hospital settings during the Christmas season (Table 1). With increased stress [3], strain, or anxiety at this time, support animals [SA] can be particularly important in providing comfort and a sense of calm (Fig. 1).

**Table 1:** Various support animals bring joy to healthcare settings at Christmas

Alpacas [4]	https://www.gloucestershirelive.co.uk/news/cheltenham-news/cosmo-alpaca-hospital-visit-gloucestershire-2353095	Gloucestershire hospital
Dogs [5, 6]	https://www.vanecovillage.com/single-post/2017/11/30/these-therapy-dogs-will-visit-you-at-home-at-christmas https://www.youtube.com/watch?v=CmCKPUZa1tY	Vancouver ecoVillage Children's Healthcare of Atlanta
Horses [7]	https://www.dailymail.co.uk/news/article-5202253/Hospital-arranges-patients-HORSE-visit-her.html	University College London Hospital
Penguins [8]	https://www.gloucestershirelive.co.uk/news/regional-news/penguins-pringle-widget-bring-happiness-7947227	Gloucestershire hospitals
Ponies [9]	https://www.bbc.com/news/av/uk-england-coventry-warwickshire-55399710	Coventry care homes
Reindeer [10]	https://www.pinterest.de/pin/why-was-reindeer-taken-round-childrens-hospital-investigation-launched-into-wellintentioned-christmas-visit-to-wards-131589620334213191/	Royal Hospital for Children Glasgow

Of course, SA are not just for Christmas. Modern roots of support animals go back a century to when Dorothy Eustis founded The Seeing Eye [11], the first guide dog school in the United States [12]. The dog breeder and philanthropist had remarkable foresight and enormous impact [13]. Her initiative helped change the public perception of how beneficial dogs and support animals could really be, especially in terms of improving the quality of life for people with disabilities. Exemplifying extreme cases, two guide dogs named Salty and Roselle [14] led their human partners, and other people, to safety through 70 floors of the burning World Trade Center and streets of rubble after the September 11 attacks in New York City. The guide dogs were honoured with awards and Roselle was posthumously named American Hero Dog of the Year 2011.

While guide dogs are specifically trained to assist people with visual impairments find their way, SA include a variety of species [15] with an increasing number of tasks to support people. One example is the use of dogs for emotional support, such as for people with mental health conditions [16]. Another example is the use of live tarantulas in exposure therapy. In effect, Eustis paved the way for a variety of – more (dogs, cats, ponies, horses, alpacas, rabbits [17]) or less (reindeer, penguins, or tarantulas [18, 19] in therapy training [19]) intuitive – animals to support people with specific needs [20].

This development is reflected in the fact that SA are now standard practice in several medical disciplines. To collate the empirical evidence on the effects of SA on different people, previous systematic reviews have focused on children, adolescents and adults as patients [21–24] and on residents in care homes [25].

Our paper focuses on another population of interest; namely, healthcare staff. Through a scoping literature review, we investigate the impact of SA on the well-being of healthcare staff within healthcare environments. Besides closing the review gap, there is another reason to target healthcare staff. This group is often overlooked in society, as was evident during the pandemic [26]. Furthermore, during the Christmas season in particular, when others spend time with their families, these dedicated people remain on duty to tend to those in need. Thus, our scoping literature review also comes from the viewpoint of “what can be done to help or support the helpers?”.

## Methods

### Scoping review

We conducted a scoping literature search of two health and life sciences literature databases (PubMed and Web of Science) using specific search terms combined with Boolean operators (Table 2) on 26 April 2023. Our research question was: “How do support animals in healthcare settings affect the well-being of healthcare staff?” We exclude legal, ethical, logistical, or administrative challenges (i.e., workload) or staff perception toward the support animal-patient relationship/ impact. Specifically, we were interested in well-being. The review was not previously registered.

**Table 2:** Search engines, string, and inclusion/exclusion criteria

Search Engines	PubMed, WoS Core Collection
**Search String**	(hospital OR inpatient OR psychiatr* OR "care unit*" OR "care hom*" OR "elderly hom*" OR hospice OR clinic* OR ward) AND ("visiting dog" OR "support dog" OR "therapy dog" OR "animal-assisted activit*" OR "animal-assisted therapy" OR "resident animal*" OR "support animal*")
**Inclusion Criteria**	1. English or German. 2. Primary Research. 3. Support animals (or similar) in healthcare units/wards/centres (not at home or for individual use) are one part of the topic of the article. 4. Measured (subjective or objective) effects on healthcare staff (e.g. mood, physical or mental health, work presence or absence *et cetera*) are one part of the topic of the article.
**Exclusion Criteria**	1. Legal, ethical, logistical, or administrative challenges only. 2. The exploration/discussion of the perception of-, effects on-, and challenges presented to, *et cetera* of healthcare staff is about bias toward the support animal-patient relationship.

After the initial step of searching the databases, article duplicates were deleted in the second step. Titles and abstracts were then screened against pre-defined inclusion criteria (Table 2) by at least two authors independently in the third step. Screening of the remaining full texts followed in the fourth step. Data were extracted and tabulated from the final pool of relevant articles. Pilot searches revealed most studies were likely to be cross-sectional, descriptive, and have low participant numbers; thus, the use of online critical appraisal tools was not pursued. At least three authors reviewed studies and contributed to their evaluation. Study findings and critical appraisal are presented in a narrative synthesis.

Popular press sources for animals visiting patients at Christmas time were identified using a non-systematic Google-based search.

### Terminology

One note on terminology: In this paper we work with the composite term “support animals”. Some authors label them support, some therapy, some facility, some visiting animals; often all have the same or overlapping roles. In all visiting cases, it appears the dogs are trained and have a handler. While there are authoritative efforts in terminology for the field going forward [20], it is difficult to provide one appropriate label in each case and to know the full extent of the role of animal in each case, especially in the psychiatric instances. Many study authors use the terms animal assisted therapy (AAT) or animal assisted intervention (AAI). There is no specific pattern or apparent way to differentiate. An umbrella term for these is animal assisted activity (AAA), which we used in the synthesis.

## Results

### Brief overview

**Fig. 2:**
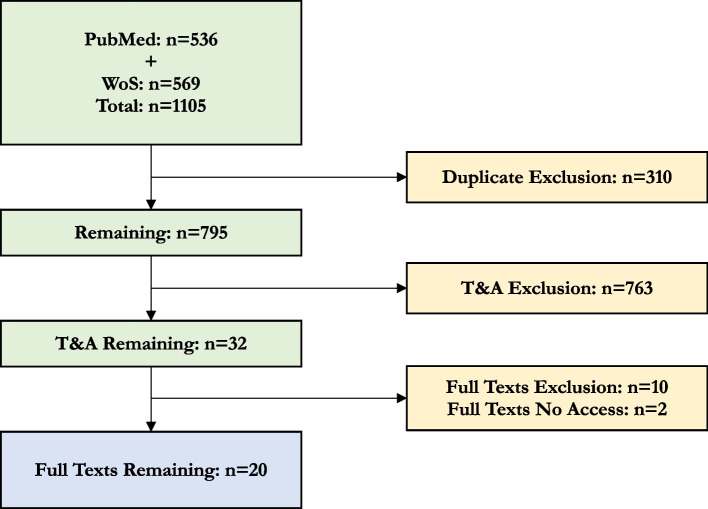
PRISMA Flow Diagram. WoS: Web of science, T: Titles, A: Abstract

From *n* = 1105 articles returned from database screening, *n* = 20 studies were deemed relevant to include in the final synthesis. The flow of articles through the screening steps is illustrated in Fig. 2. Extracted data is presented in Table 3. The table and synthesis are organised as follows: studies with biological measures (*n* = 3), longitudinal survey studies with analyses (*n* = 5), cross-sectional survey studies with analyses (*n* = 2), and cross-sectional survey studies with descriptive statistics (*n* = 10). In our synthesis, we do not stratify by recency, animal species, or country for the following reasons: All articles were published since 2002. Thirteen of these were published within the last 4 years. All studies involved dogs except for one study that involved cats [27]. All settings were located in the USA, Australia, and Europe.

**Table 3:** Overview of included studies (all studies involve dogs except for one involving cats which is labelled)

Author (Year)	Study	Population	Results
***Studies with biological measures***			
Kline (2020) [28]	Repeated biological measures Colouring mandala vs staff AAT vs no intervention	AAT: *n* = 43, mean age 31 years Colouring: *n* = 40, mean age 32 years Control: *n* = 39, mean age 33 years Staff: nurse, physician Setting: Emergency department, safety net hospital	-Apparent differential effects for subjective stress and cortisol -Cortisol lower at end of shift for AAT and colouring compared to control -Frequent disappointment expressed when assigned to colouring rather than AAT
Machová (2019) [29]	Repeated biological measures Break with/without staff AAT and no break	*n* = 20 Staff: nurse Setting: Departments of physical medicine and rehabilitation, internal medicine and long-term care, military hospital	-Decrease in cortisol after AAT only but not for nurses in the physical medicine and rehabilitation departments
Barker (2005) [30]	Repeated biological measures Breaks with/without staff- and patient therapy dog	*n* = 20, age (mean) 38.6 years Staff: nurse, physician, therapist Setting: Inpatient services, academic medical centre	-Cortisol decline appears equivalent for breaks of 20 min + dog, 5 min + dog, and 20 min-dog -No apparent changes in noradrenaline, adrenaline, lymphocyte proliferation, or salivary IgA
***Longitudinal studies with survey analyses (no biological measures)***			
Gerson (2023) [31]	Survey Pre- vs post staff AAT	*n* = 149 Staff: Any hospital staff could participate Setting: Academic children’s hospital	-Feelings of reduced tiredness, discomfort, pain, negative emotions -Feelings of increased comfort, energy, positive emotions
Etingen (2020) [32]	Surveys, semi-structured interviews Pre vs post staff AAI	Pre: *n* = 22 Post: *n* = 16 Staff: nurse, physician, assistant, ‘others’ Location: Clinic	-Majority liked AAI, improved mood, stimulating social interaction -No change in perception of burnout except for less exhaustion after interaction with patients
Brown (2020) [33]	Survey, open-ended question Pre vs post AAA	Adult unit: *n* = unclear (16 or 20), mean age 33.7 years Adolescent unit: *n* = 8, mean age 41 years Setting: Psychiatric inpatient units, teaching hospital	-Reduced negative moods in adult unit, no change in adolescent unit
Ginex (2018) [34]	Survey Pre vs post patient AAT	*n* = 41 Staff: nurse, carer, technical/assistant, ancillary Setting: Adult surgical oncology unit, hospital	-Lower stress, sense of happiness and hopefulness, pleasant distraction -No change in burnout, compassion
Marcus (2012) [35]	Survey Pre vs post accompanying patient with AAT	AAT: *n* = 7, age (mean) 31.9 years No AAT: *n* = 22, age (mean) 39.8 years Staff: nurse, physician, assistant, admin, therapist, management Setting: chronic pain, university outpatient clinic	-Fatigue, stress, and positive affect improved in AAT -Positive comments: 100% of staff enjoyed dog visits -Improved calmness only with no AAT
***Cross-sectional survey analyses***			
Jensen (2021) [36]	Survey Working with/without AAT	*n* = 130, age (mean) 37 years Staff: Paediatric healthcare professionals Setting: children’s hospital	-Higher perceived personal accomplishment, more positive job descriptions, lower intention to quit, positive emotions, better-perceived mental health, and less depression -No association with emotional exhaustion, perceptions about co-workers, workplace social support, or anxiety
Moody (2002) [37]	Survey Expectation vs experience of patient AAT	Expectation: *n* = 115 Experience: *n* = 45 Staff: nurse, physician, technical, carer, admin Setting: Paediatric ward, children’s hospital	-Non-medical staff more positive than medical staff -Work environment rated higher by Experience vs Expectation -Program acceptance rated higher by Experience vs Expectation
***Cross-sectional surveys, descriptive***			
Caton (2021) [38]	Survey Staff AAT	Setting: Emergency department, intensive care, burns, trauma, and high acuity units, hospital	-High agreement of positive interactions, stress reduction, increased morale, support for continuation, feeling cared for by organization, and improved unit environment
Pruskowski (2020) [39]	Survey Patient AAT	*n* = 23 Staff: Rehabilitation staff Setting: Burn centre, military hospital	-Most reported improved mood, would like to see or work dogs again, several commented that therapy dogs were great stress relievers, no negative comments
Machová (2020) [40]	Survey Patient AAI	*n* = 36, age (mean) 44.6 years Staff: healthcare and social workers Setting: Nursing or retirement home, household hospice	-Overwhelmingly positive perception of AAI from staff regarding stress, fears, bringing pleasure
Wagner (2019) [27]	Interviews Cats living on the ward	*n* = 17, mean age 40.6 years Setting: Acute psychiatric wards, university psychiatric clinic	-All staff had positive feelings about the cat, most reported positive impact on work satisfaction and emotional well-being
Uglow (2019) [41]	Survey Patient AAI	*n* = 82 Staff: nurse, physician, therapist, admin, radiographer, technical, ‘others’ Setting: Surgical, medical, high dependency, intensive care, and day wards, university children’s hospital	-None of the staff had any concerns regarding the program, no reports of disruption -100% considered the AAI program as very worthwhile
Rothschild (2019) [42]	Survey, interview, focus group Communal pet living on site	*n* = 12 Staff: nurse, psychologic, social Setting: Community care unit for mentally ill	-Helpful to recovery, dog provides relaxing and peaceful atmosphere and has a calming effect, motivates to do more exercise -Some concerns that staff/residents may have fears
Fodstad (2019) [43]	Survey Patient AAA	*n* = 33 Staff: nurse, physician, technical, therapist Setting: Paediatric behavioural health unit, children’s hospital, academic medical centre	-Majority report positive effect on themselves and their work, including stress relief
Abrahamson (2016) [44]	Pilot interviews about AAI dogs in reception, waiting rooms, emergency department, and surgical units	*n* = 9 Staff: nurse, technical/assistant, volunteer Setting: Medical and surgical community hospital	-Most excited to meet dog, made effort, all sought physical contact, felt more relaxed, happy, less stressed -One respondent had concerns regarding infections and would prefer limited access of dog to certain areas
Bibbo (2013) [45]	Survey Patient AAA	*n* = 34 Setting: Regional outpatient cancer centre	-Perceptions to AAA were mostly positive
Caprilli (2006) [46]	Survey Patient AAA	*n* = 52 Staff: nurse, physician, assistant Setting; Paediatric ward, children’s hospital	-54% think animal program can benefit them -16% afraid of dog bites or disease transmission

*AAA* Animal assisted activities, *AAT* Animal assisted therapy, *AAI* Animal assisted intervention

Healthcare settings were mostly hospitals or medical centres, seven of which involved children’s hospitals or paediatric wards (one of which was a psychiatric ward for adolescents) [31, 33, 36, 37, 41, 43, 46]. Two studies specify community care/hospice [40, 42]. Apart from the two latter studies, all studies at least implied assessment of nurses and physicians (thirteen explicitly mentioned this). Some studies also considered allied and ancillary healthcare staff [30–41, 43, 44]. In total, ~ 1,000 members of healthcare staff participated across the twenty studies.

Regarding exposure/intervention, *n* = 6 studies considered reaction to staff AAA, *n* = 11 studies considered reaction to patient AAA, *n* = 3 studies considered reaction to communal animals [27, 42, 44]. Regarding outcomes, perceptions, attitudes, concerns, emotional well-being, fatigue, burnout, physical discomfort and pain, cortisol, noradrenaline, adrenaline and IgA were assessed.

### Biological measures

**Fig. 3:**
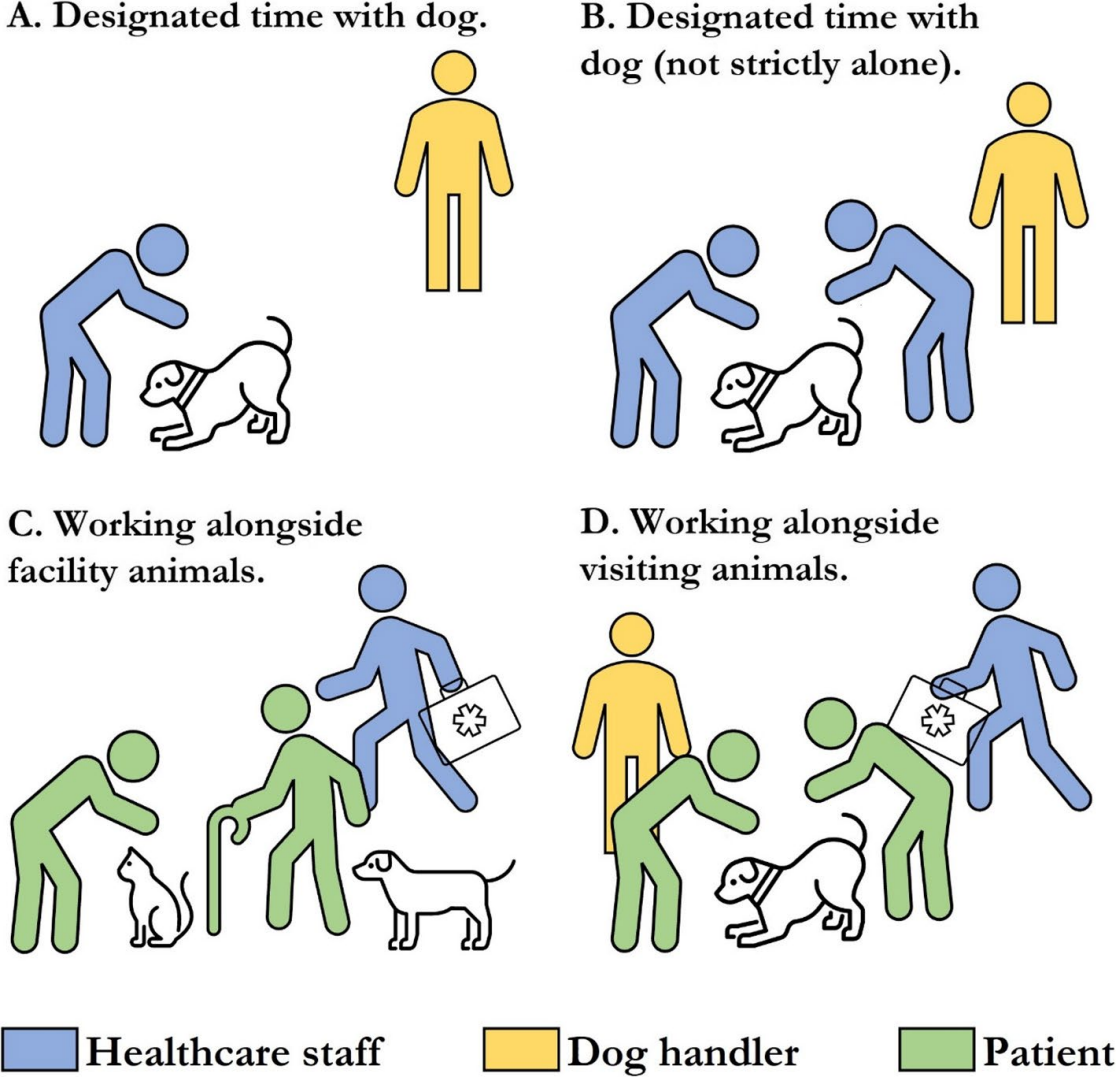
Healthcare staff interactions with support animals that were investigated. **A** Designated time alone with dog (with handler present) [28, 29]; **B** Designated time with dog (with handler and possibly others present) [30–35]; **C** Working alongside facility (full-time) animals including dogs and cats [27, 36, 42]; **D** Working alongside dogs visiting patients

Three studies assessed cortisol in serum or saliva [28–30]. Machova et al. (2019), using a within subjects design, observed decreased cortisol in some staff after taking a break with the dog but not after taking a break without the dog or after no break (Fig. 3) [29]. Baseline cortisol was measured at the same time every day (10:00am) and 50 min later (within which the break occurred). The reduction was observed in *n* = 9 nurses working at the department of internal medicine and long-term care but not in *n* = 11 nurses working in physical medicine; the authors put forward as explanation that these participants started with already low cortisol values. Barker et al. (2005) assessed cortisol at several time points following 5 min rest with dog, 20 min rest with dog, 20 min rest without dog with the interventions starting at either 1:00 pm or 3:00 pm. They observed decreased cortisol after at least 45 min in all three groups but no changes in noradrenaline, adrenaline, salivary IgA, or lymphocyte proliferation [30]. Decreased cortisol was observed at 15 min post intervention for both 20 min rest with and without dog but not 5 min with dog. Of course, the time since beginning of intervention is different for these measurements (e.g., 20 min + 15 min vs 5 min + 15 min); thus, conclusions about group differences cannot be made. Kline et al. (2020) assessed cortisol across a work shift that included either 5 min with dog (*n* = 43), 5 min colouring a mandala (*n* = 40), or no intervention (*n* = 39) [28]. Using a mixed methods repeated measured ANOVA, they observed time x group interactions suggesting greater decrease in cortisol at the end of shift in the 5 min with dog group compared to other groups. Of note, differential effects for subjective stress (and compared to cortisol) were observed insofar as there was no clear effect on subjective stress as measured with the modified Perceived Stress Scale but there was when using a visual analogue scale. Also of interest, a potential rise in subjective stress associated with colouring was observed, possibly attributable to the frequently expressed disappointment when assigned to colouring rather than the dog intervention.

### Longitudinal studies with survey analyses

All five studies used a pre- vs post- design. Two studies assessed responses to AAA for staff [31, 32] and three studies to AAA for patients [33–35]. Regarding staff-specific dogs, Gerson et al. (2023) examined the effect of a “Medical Dog Office Hours Program” of maximum 10 × 1-h sessions over 1–2 months and Etingen et al. (2020) adopted a similar procedure with a maximum of 20 × 1-h sessions over 3 months [31, 32]. In both cases frequency and duration of participation differed by participant availability. Also in both cases, the impact of staff AAA was considered beneficial with improved mood and decreased tiredness. In the latter study, there was no clear effect on perception of burnout except for less exhaustion when dealing with patients [32]. The latter program was also rated as positive (not asked in the former) [32].

Regarding responses to AAA for patients, all three studies observed improved mood in staff in different adult units compared to baseline [33–35]. Ginex et al. (2018) also observed decreased feeling of stress but no change in feelings of burnout or compassion in an oncology unit [34]. Marcus et al. (2012) also observed improvements in feelings of stress and fatigue in a chronic pain unit [35]. In contrast to their findings on an adult psychiatric unit, Brown et al. (2020) did not observe a change in mood on an adolescent psychiatric unit [33]. Ginex et al. (2018) took post-measurements at least six weeks after full implementation of an AAA program that involved visits on four days per week [34]. Marcus et al. (2012) conducted a 2-month long study of AAA visits to their waiting room for 2-h on 2 days per week [35]. Brown et al. (2020) conducted a 4-month long study of weekly dog visits. In all cases, it is unclear how much interaction staff had with the dogs prior to starting the study or exact the time difference between pre- and post- measurements for each member of staff [33]. In the study by Marcus et al. [35], all staff reported enjoying dog visits.

### Cross-sectional surveys—analyses

Two studies conducted cross-sectional survey analyses. Moody et al. (2002) compared expectation vs experience of AAA for patients and Jensen et al. (2021) compared working with vs without a facility dog for patients [36, 37]. Both studies were conducted at a children’s hospital. Moody et al. (2002) found that the work environment and program acceptance was rated higher by those who experienced AAA for patients (measured 12 weeks after program start; unclear how long staff actually experienced AAA) [37]. Jensen et al. [36] found that those staff working with a facility dog had higher perceived personal accomplishment and job satisfaction and better mood. There were no differences in feelings of fatigue or perceptions of co-workers, workplace support, or anxiety [36].

### Cross-sectional surveys—descriptive

Comprising ten studies, this was the most common study design. One study concerned AAA for staff [38]. Two studies included communal animals (psychiatric ward with cats and community care for mentally ill with dog) [27, 42]. The remaining seven studies concerned responses to patient AAA [39–41, 43–46]. All studies indicate positive perception or experience of AAA. In particular, and irrespective of whether AAA was primarily directed towards staff or patients, there was agreement that AAA was beneficial against stress [38–40, 42–44]. In some studies, staff members expressed excitement about the opportunity to interact with dogs [39, 44]. As examples of widespread agreeableness with AAA: Caton et al. (2021) find over 90% of staff participants acknowledged a positive impact on staff morale, program satisfaction, and stress reduction and 96% expressed a desire for the program to continue [38]; Pruskowski et al. (2020) find 95% of rehabilitation staff consider their mood is better [39]; Machova et al. (2020) find 92% of staff participants consider that the dogs provide them with emotional support [40]; Wagner et al. (2019) find 100% of staff participants had positive feelings about the cats and 84% reported a positive impact on their work satisfaction [27]; Uglow et al. (2019) find 100% of staff participants consider AAA to be very worthwhile [41]. The lowest observed agreement or benefit of AAA was still 54% in the study by Caprilli et al. (2006) [46]. Indeed, in one study, staff even also reported missing the dog when it was not present [44]!

There were few negatives and in only a few studies. Specifically, some individual expressed concerns regarding fear of dogs, potential dog bites, cleanliness issues, disease transmission, and added stress [42, 44–46]. Caprilli et al. (2006) reported that 16% of staff expressed fear of dog biting or disease transmission, but there was no change in infection rates after the introduction of dogs, and no reports of biting or other problems with the dogs [46]. This is not to say that these are the only studies that asked about negative experiences or concerns. For instance, Machova et al. (2020) found no concerns regarding contamination of beds or rooms and this study was conducted in a nursing/retirement home and home hospices following AAA visits [40].

## Discussion

In regards to our research question “How do support animals in healthcare settings affect the well-being of healthcare staff?”, our overall answer, based on the results of 20 included studies (Table 3; 19 studies with dogs and 1 study with cats) is that AAAs appear to be generally well received by staff. This appears to be the case regardless of whether the AAA is primarily intended for staff or for patients, even though cross-sectional survey studies typically include few participants (in some groups as few as *n* = 7). It is difficult to conclude that the AAA leads to a reduction in cortisol levels among staff, as this was not observed among all staff in one study and some differences between cortisol levels and subjective stress were observed in another [28, 29]. Negative impact on staff well-being was not observed as a pattern. Regarding more unique animals that may visit healthcare settings at Christmas in particular, there are no studies of impact on healthcare staff well-being.

Following the early use of dogs for visually impaired or blind people, SA are now increasingly used to assist, for example, people with mental health conditions, including anxiety, depression, and stress and can be especially beneficial for people who struggle with social isolation or other forms of emotional distress. More generally, SA can provide comfort, security, and companionship that can be difficult to find elsewhere [47]. In particular, SA can also help individuals with PTSD [48], autism, and other conditions by assisting them with everyday tasks and helping to manage symptoms. Such aspects may shape staff expectations of AAA. Furthermore, positive impressions such as by SA visiting healthcare facilities around Christmas (Table 1) convey their additional emotional value. In line with the literature demonstrating beneficial impact of SA on well-being of patients, we find SA may also benefit the well-being of healthcare staff.

Importantly, although the use of SA can be beneficial, there are also concerns – medical and ethical – associated with their use. For instance, contact with SA can be problematic for people with allergies, with a suppressed immune system and/or fears of animals – see study information above Table 3). Limited sample sizes and voluntary participation and animal interactions in the reviewed studies may not have included few (if any) of these individuals, causing selection bias. Indeed, such selection was actively pursued in some studies: “Women who had a positive attitude to dogs and who agreed to participate in this study were deemed suitable candidates” [29]. Of course, we should not force SA interaction on workers. Beyond the reviewed studies, there is – for instance – concern that some SA may not be adequately trained or socialized, leading to safety concerns [49]. Hospitals and healthcare staff may have to assess the individual needs of each patient (and member of staff) to decide on SA on a case-by-case basis. In addition, animals must be trained, certified and properly supervised during their visits to ensure the safety and well-being of all patients, staff and the animals themselves. This may be more important for more unusual animals that may visit at Christmas time.

The latter requires qualification: As for the empirical “1^st^” in our review of a baby elephant visiting a children’s ward in the 1950s, the keeping of large animals in circuses has been frowned upon and often banned for some time now. The additional presence of a clown might have posed an extra burden for certain individuals, such as children and clinicians, due to potential fear and distress associated with clowns [50, 51]. While the prevalence and impact of coulrophobia (the fear of clowns) [1] is not clear, what is clear is that numerous children (and adults) are terrified of dogs. Decades ago, a 12-year-old boy exemplified things to be afraid of as “Wild animals, fierce dogs and cats, and snakes” [52]. Dogs [53], for instance, may, and will(!), be a source of fear to several people – young and old – in healthcare settings. It thus follows for organizing less or more unusual animals to visit healthcare settings (Table 1), that people (be they patients or residents or staff in hospitals or care facilities) and animals must be carefully selected before possible interactions.

Importantly, while humans are of course critical, ethics must not stop at anthropocentric viewpoints [17]. The health of the animals is important, too. A necessary recommendation is to regularly monitor the physical and mental health of SA. Such assessments should be conducted at all stages of AAA, i.e. before, during, and after the human-animal interactions. To ensure the health and welfare of the animals, a veterinarian who is familiar with both the biology and behaviour of the SA is preferably consulted. For example, a prerequisite for the ethical treatment of SA is a distraction-free environment. A recent study – albeit in a zoo setting – on the welfare implications of various species (giraffe, tapir, lama) conveyed that Christmas Evening events had no obvious negative impact on the animals [54]. In principle, however, any events at unusual times – such as possibly for pets at Christmas in healthcare settings – should allow animals some leeway to feel comfortable with the tasks. Finally, we should also recognize the various roles non-human animals may have, including very unusual ones [55].

Overall, it is both possible and can be beneficial to incorporate SA in hospital [56, 57] and other healthcare settings, including during the Christmas holidays. As shown in Table 1, carefully selected animals can provide emotional support to – carefully selected – patients and help reduce stress and anxiety during hospital stays [58]. As shown in Table 3, the same appears to hold for healthcare staff. To this end, many hospitals have programs that allow certified SA to visit patients, and some hospitals even have their own SA living on site. During the Christmas season, hospitals may organize special events or programmes where SA contribute to activities around the holidays, which may be particularly important and beneficial for healthcare professionals who feel particularly isolated during the festive season.

In summary, support animals are useful, relatively safe [49], and necessary in countless cases for people with various problems and support needs in various forms. While our scoping review does not show how exactly AAA benefits staff, it does identify relevant positive effects and avenues of research. With appropriate security precautions, gaps in hard-fact-evidence should not deter us – especially at the festive season – from encouraging work with SA that provides warmth, empathy, comfort and more in healthcare settings. We conclude that the presence of, and interaction with, support animals in healthcare settings can be a promising health resource which should not be left untapped and warrants systematic research. Importantly, support animals are not just for Christmas, but can provide comfort all year round .

## Data Availability

N/a.
